# Endoscope-assisted right coronal suturectomy

**DOI:** 10.3171/2021.1.FOCVID20130

**Published:** 2021-04-01

**Authors:** Michael M. McDowell, Robert Kellogg, Jesse A. Goldstein, Taylor J. Abel

**Affiliations:** Departments of 1 Neurological Surgery and; 2 Plastic Surgery, UPMC Children's Hospital of Pittsburgh, Pennsylvania

**Keywords:** craniosynostosis, endoscopic, coronal, sagittal, spring-assisted

## Abstract

Endoscopic suturectomy combined with supplementary techniques such as spring-assisted expansion and cranial molding helmets for the correction of craniosynostosis is growing in popularity due to the reduced scar burdened, decreased morbidity, and reduced overall cost. The authors present their technique for the correction of isolated coronal craniosynostosis. The use of dedicated endoscopic tools and lit endoscopes permits enhanced visualization and technical ability, particularly at the distal portions of the suturectomy, and may reduce operative time and cerebrospinal fluid leak risk.

The video can be found here: https://vimeo.com/515401366.

## Transcript

This is Michael McDowell at University of Pittsburgh Children's Hospital. We will present today a case of single-suture coronal craniosynostosis.

Indications for this approach are single-suture pathology and a strong preference of patients age 3 months or younger. Patients with relatively mild deformity or with comorbidities increasing the risk of prolonged surgeries or higher blood loss are considered up to the age of 6 months.

In terms of preoperatively planning, we make a 2- to 3-cm incision parallel to the affected suture midway along its course. Positioning is supine.

**0:58 Case Highlight**. This case is of a 3-month-old boy with progressive harlequin deformity and right anterior plagiocephaly. He was otherwise developmentally and neurologically normal and was found to have isolated right coronal craniosynostosis. A endoscopic suturectomy with postoperative helmeting was offered.

**1:15 Preoperative**. Here is a preoperative photo taken with consent of the family.

As you can see here, the suture is mapped out and an incision planned at about the halfway point. The incision is made sharply through galea, which is dissected way from the pericranium. After hemostasis is obtained, the pericranium is tattooed using cautery in order to map out the course of a 1-cm suturectomy. A standard operative ruler has a width of 1 cm and thus is useful as a stencil initially. It is then removed and the suturectomy is marked out laterally along the coronal suture by retracting the scalp.

**1:57 Burr Hole**. A burr hole is then made directly above the coronal suture and then extended laterally with rongeurs toward the sphenoid wing. As a general rule, we prefer to perform the suturectomy first in the direction that is most dependent in order to prevent obscuration from bloody rundown later in the case. Standard Kerrison rongeurs are helpful initially, but once under the scalp line we transition to endoscopic tools.

**2:27 Sphenoid Is Visualized**. Once the greater wing of the sphenoid is visualized, the lighted endoscope can be inverted to allow for visualization of its inferior ridge, which is dissected and then bitten down with a straight through-cut. We flatten the greater wing and use the visibility of the frontal and temporal poles as a marker of depth. The thickest portions of the wing are often more easily removed using a pituitary rongeur, which has greater strength but is not a cutting instrument. The frontal and parietal bones are palpated for mobility through the scalp.

**3:11 Medial Coronal Suture Is Visualized**. The medial coronal suture is then visualized and the location of the anterior fontanelle confirmed visually with palpation. The majority of this suturectomy can easily be performed with a Leksell or needlenose rongeur. The fontanelle itself can be released using a pituitary rather than a sharper through-cut in order to avoid durotomy. The bones are again palpated to confirm good mobility. A galeal closure with vicryls, followed by a dermal closure with plain gut suture, is performed. A drain is typically not required in endoscopic cases.

**4:13 Postoperative Course**. Postoperatively, the patient tolerated the procedure well and was discharged in good condition on postoperative day 1.

Helmeting fitting was 1 week postoperatively. Helmeting was initiated 2 weeks postoperatively and continued for 1 year. Here is a postoperative photo of the patient on long-term follow-up, as well as a comparison photo of preoperatively and postoperatively.

**4:23 References** ^1–5^

## Author Contributions

Primary surgeon: Abel, Kellogg, Goldstein. Assistant surgeon: Abel, McDowell, Goldstein. Editing and drafting the video and abstract: McDowell. Critically revising the work: Abel, McDowell. Reviewed submitted version of the work: all authors. Approved the final version of the work on behalf of all authors: Abel. Supervision: Abel, Goldstein.
